# Loop modeling: Sampling, filtering, and scoring

**DOI:** 10.1002/prot.21612

**Published:** 2007-08-29

**Authors:** Cinque S Soto, Marc Fasnacht, Jiang Zhu, Lucy Forrest, Barry Honig

**Affiliations:** Howard Hughes Medical Institute, Center for Computational Biology and Bioinformatics, Department of Biochemistry and Molecular Biophysics, Columbia UniversityNew York, New York 10032

**Keywords:** DFIRE, OPLS force field, loop ensembles, loop closure algorithm, LoopBuilder, LOOPY

## Abstract

We describe a fast and accurate protocol, LoopBuilder, for the prediction of loop conformations in proteins. The procedure includes extensive sampling of backbone conformations, side chain addition, the use of a statistical potential to select a subset of these conformations, and, finally, an energy minimization and ranking with an all-atom force field. We find that the Direct Tweak algorithm used in the previously developed LOOPY program is successful in generating an ensemble of conformations that on average are closer to the native conformation than those generated by other methods. An important feature of Direct Tweak is that it checks for interactions between the loop and the rest of the protein during the loop closure process. DFIRE is found to be a particularly effective statistical potential that can bias conformation space toward conformations that are close to the native structure. Its application as a filter prior to a full molecular mechanics energy minimization both improves prediction accuracy and offers a significant savings in computer time. Final scoring is based on the OPLS/SBG-NP force field implemented in the PLOP program. The approach is also shown to be quite successful in predicting loop conformations for cases where the native side chain conformations are assumed to be unknown, suggesting that it will prove effective in real homology modeling applications. Proteins 2008. © 2007 Wiley-Liss, Inc.

## INTRODUCTION

Template-based protein structure prediction is being increasingly used in a variety of biological applications.^1^–^3^ The most accurate models are obtained in cases where a template can be found in the Protein Data Bank (PDB)^4^ with a high level of sequence similarity to a query protein. This corresponds to comparative or homology modeling. However, in the general and increasingly common situation, it is appropriate to splice together more than one template or structural fragment to create a full model for a protein. In either case there will inevitably be parts of the structure that cannot be modeled based on known structures or for which an appropriate template, even if it exists, cannot be identified. In such cases, it is necessary to use *ab initio* methods to predict the structure of the region in question. These regions can correspond to long insertions or deletions or to short loops that connect secondary structure elements. The latter situation is quite common. It arises even for relatively straightforward cases of homology modeling simply because homologous proteins often have loops of different lengths so that the template and query loop conformations will often be different.

The loop modeling problem has a long history and the interest in its solution goes beyond the prediction of small insertions and deletions in homology models. Specifically, many of the problems encountered in loop modeling are the same as those encountered in the larger problem of protein structure prediction; it is the scales of the two problems that are very different. Both problems require extensive conformational sampling and refinement and both depend on the quality of energy or scoring functions used to identify stable conformations. In both cases, the standard test of a method is in its ability to identify a native-like conformation, usually on the background of a large number of incorrect conformations. Indeed, it can be argued that the ability to predict loop conformations is a prerequisite for predicting and refining protein structure. Moreover, there are many cases where loops undergo functionally significant conformational changes whose understanding in atomic detail is of particular interest.^5^–^7^ One might reasonably expect that any approach used to study such changes would first be tested on straightforward loop modeling problems.

Our goal in this work is to develop a loop modeling procedure that is both computationally efficient and accurate in its ability to predict native-like conformations. To this end we first evaluate approaches currently used at different stages in the loop prediction process. We begin with a summary of the recent literature with goal of evaluating the current state of this field. It should be recognized that most studies of the loop prediction problem assume that the conformation of the rest of the protein, except for the loop, is known. This does not correspond to a realistic modeling situation but it does provide a well-defined control problem which can be used to evaluate different methods.

Recent advances in *ab initio* loop modeling have reached the point where predicting the conformations of loops containing up to seven residues can usually be done with considerable accuracy.^8^,^9^ Database approaches^10^,^11^ based on the extraction of loop structures from the PDB have not, in general, reached the same level of accuracy although significant progress has recently been reported.^10^ Moreover, remarkably accurate predictions can be made for longer loops if the crystal contacts are taken into account and if extensive conformational sampling is used (e.g., <1.5 Å RMSD from native for loops 11–13 residues in length).^12^ This is an important result because it shows what is possible given enough constraints. It also demonstrates that there may be inherent limits to the accuracy of loop modeling for the simple reason that the conformation in the reference crystal structure may be determined in part by packing constraints. Still, the steady progress that has been reported suggests that we may have not reached these limits. Table I reports prediction accuracy taken from the literature, in chronological order, of methods that have been reported in the past few years. The methods were not applied to the same loop set, so that caution must be exercised in making direct comparisons. Note that the most accurate results are from the papers of Jacobson *et al*.^9^ and Zhu *et al*.,^12^ in which crystal contacts were taken into account. It should be pointed out that Rohl *et al*.^13^ predict the conformations of all side chains in a protein in their procedure whereas other methods use the experimental conformations for all side chains except those in the loop.

**Table I:** Loop Prediction Accuracy of Published Methods

RMSD (Å)						
Loop length	Modeller^a^	LOOPY^b^	RAPPER^c^	Rosetta^d^	PLOP^e^	PLOP II^f^
8	2.5	1.45	2.28	1.45	0.84	NA
9	3.5	2.68	2.41	NA	1.28	NA
10	3.5	2.21	3.48	NA	1.22	NA
11	5.5	3.52	4.94	NA	1.63	1.00
12	6.0	3.42	4.99	3.62	2.28	1.15
13	6.5	NA	NA	NA	NA	1.25

a Data taken from Figure 9 of Fiser *et al*.^14^

b Data taken from Table I of Xiang *et al*.^8^

c Data taken from Table III of de Bakker *et al*.^15^

d Data taken from Tables IV and VV of Rohl *et al*.^13^

e Data taken from Table IV of Jacobson *et al*.^9^

f Data taken from Table II of Zhu and Pincus *et al*.^12^

The methods summarized in Table I are quite different from one another in detail, but most methods begin by sampling a large number of sterically feasible backbone conformations with side chains added in a separate step. In contrast, the loop prediction program in Modeller constructs and samples loop conformations, including side chains, with a bond-scaling and relaxation method that uses a combination of conjugate gradient minimization and molecular dynamics with simulated annealing.^14^ The LOOPY algorithm^8^ (see also later) is based on a modified version of the Random Tweak algorithm^16^ that carries out loop closure while avoiding steric clashes. RAPPER samples conformational space using a fine-grained set of φ ϕ, states while avoiding steric clashes.^17^ Rosetta uses a combination of database-derived fragments of protein structure from the PDB and assembles them with a Monte Carlo procedure followed by simulated annealing.^13^ PLOP samples conformational space using a systematic dihedral-angle based build-up procedure that constructs fragments from the N-terminal and C-terminal stems that meet in the middle.^9^,^12^

Each of the methods in Table I relies on the use of a scoring function aimed at selecting the most energetically favorable conformations from the ensemble of loops that are generated. Here again there is considerable diversity in the approaches that are taken. The LOOPY algorithm uses a simple heuristic scoring function that accounts approximately for van der Waals interactions, hydrogen bonding, and hydrophobicity, and includes a “colony energy” term that attempts to account for conformational entropy.^8^ The RAPPER algorithm uses the RAPDF^18^ statistical potential to filter loop ensembles followed by all-atom energy minimization on a subset of loops from the ensemble using the AMBER force field with a generalized Born solvation term.^15^ The combination of *ab initio* loop generation with subsequent molecular mechanics energy minimization has been used for some time in loop modeling.^19^,^20^ PLOP uses the all-atom OPLS force field with a generalized Born solvation term. Rosetta uses its own scoring function which has both statistical and physico–chemical features.^21^

The computational demands posed by the different methods also vary greatly. LOOPY makes a full prediction for an eight residue loop in about 20 min on a 1.3 MHz processor while the other methods require hours or more. In contrast PLOP is extremely accurate but very slow, taking hours to days for a comparable problem.

A number of conclusions emerge from an analysis of the results summarized in Table I. The PLOP results show that truly high-quality results are obtainable given an accurate molecular mechanics force field and sufficient computer time to carry out extensive conformational sampling. However, we do not know how well this procedure would work if crystal contacts were not taken into account. Moreover, the procedure is quite slow and becomes increasingly inefficient for longer loops. LOOPY's heuristic function appears to work quite well but the quality of the results also degrades for long loops and its use of an approximate heuristic potential function limits its ultimate accuracy. The Rosetta procedure is hard to compare with other methods because it repacks side chains on the entire protein (not just the loop residues) so that its results as reported in Table I are for a harder problem than attacked by the other algorithms (see, however, later). Finally, RAPPER appears to be less accurate than the other procedures but its use of a statistical potential allows a fast conformational energy evaluation that should prove increasingly useful for predictions on longer loops.

The goal in this work is to develop a loop prediction protocol that approaches the level of accuracy obtained by PLOP but that is computationally efficient. To this end we first consider a number of loop closure procedures that have recently been reported in the literature and evaluate them in terms of their ability to generate sterically reasonable native-like loop conformations. We then test the ability of statistical potentials to identify native-like conformations guided in large part by recently reported successes of the DFIRE potential.^22^,^23^ On the basis of our results, we describe a loop prediction protocol (LoopBuilder) that is similar in principle to the one used in RAPPER, but is different in the details. Specifically, we use the LOOPY program to generate a starting ensemble of sterically reasonable conformations including side chains, DFIRE to select a subset of these conformations, and, finally, an all-atom energy minimization. The results that we obtain improve upon those reported in Table I (except for those obtained from PLOP) and the calculations do not involve significant computational cost. Moreover, the protocol is modular thus allowing for the introduction of new algorithms and scoring functions at any stage of the process.

## MATERIALS AND METHODS

### Loop datasets

Much of our analysis is carried out on loops used in the study of Jacobson *et al*.^9^ which is a filtered set taken from 8–12 residue data sets compiled by Fiser *et al*.^14^ and Xiang *et al*.^8^ We also used a set of 11,12, and 13-residue loops taken from the study of Zhu *et al*.^12^ Both Jacobson *et al*. and Zhu *et al*. filtered out loops whose structures were crystallized at a nonstandard pH, contained any atom in the loop region within some 4.0 Å of any neutral ligand or 6.5 Å of any metal ion and whose average β-factor summed over N, Cα, C, O, Cβ was larger than 35 Å^2^. In total, we considered 63 eight, 56 nine, 40 ten, 54 eleven, 40 twelve, and 40 thirteen-residue loops.

In all cases, we used the global root-mean-square deviation (RMSD) measure using the N, Cα, C, and O atoms to compare the structural similarity of a predicted loop conformation with the native loop conformation. The global RMSD is measured after optimal superposition of the body of the protein (i.e., all backbone heavy atoms excluding those atoms belonging to the loop).

### Loop closure methods

The algorithms compared in this section include cyclic coordinate descent (CCD)^24^, Wriggling^25^, PLOP-build^9^ (version 4.0), LOOPY, and two algorithms used in LOOPY, Random Tweak and Direct Tweak. We used in-house implementations of CCD and Wriggling (implementing published algorithms and convergence criteria), while the other programs either originated in our lab (LOOPY, Random Tweak and Direct Tweak) or were obtained from their authors (PLOP-build^9^). In the case of CCD, we modified the published algorithm so that closure conditions used for the C-terminal stem were changed from the (N, Cα, C) atoms to (Cα, C, O) atoms. This was necessary to facilitate comparisons with the other algorithms. CCD, Wriggling, and Random Tweak generate closed loops without accounting for steric overlaps as does PLOP-build as used here (steric clash filter turned off). The Random Tweak algorithm is the one used in LOOPY^26^ which avoids the chirality issues that were present in Shenkin and Levinthal's original implementation.^16^ Random Tweak generates loop conformations that are open at one end and then closes them by making small changes to Φ/Ψ angles of the loop to enforce distance constraints between corresponding atoms between the flying and fixed stems. This is done using an iterated Lagrange multiplier method that satisfies distance constraints imposed by the stem residues.

“Direct Tweak” is similar to Random Tweak but also includes a nonbonded energy term in the iterated Lagrangian formulation that simultaneously enforces distance constraints while avoiding steric clashes.^8^,^26^ Direct Tweak differs from the other algorithms used here in that its minimization procedure accounts for steric interactions between loop atoms and atoms in the rest of the protein. The LOOPY algorithm^8^,^26^ uses both Random Tweak and Direct Tweak. Closed loops are generated with Random Tweak and are then filtered for steric clashes with a heuristic scoring function. Side chains are then added with a modified version of the SCAP^27^ algorithm and the loop conformations that survive the filter are energy minimized with Direct Tweak. This is the method that is used in LoopBuilder (see later) but in order to allow comparisons with the other loop closure methods, in this section we skip the side chain addition step. We refer to this algorithm as LOOPY_bb_ where ‘bb’ indicates backbone atoms only. LOOPY_bb_ thus involves loop closure with Random Tweak followed by minimization in torsion angle space of backbone atoms with Direct Tweak. Here we compare Direct Tweak and LOOPY_bb_ with loop closure methods such as CCD, Wriggling, Random Tweak, and PLOP-build that do not account for steric hindrance during the closure procedure.

A loop conformation was considered successfully closed if the RMSD between the Cα and C atoms at the C-terminus of the open loop conformation (i.e., flying stem) and the corresponding atoms on the fixed stem was less than 0.25 Å. Since PLOP-build generates fragments starting from the N-terminal and C-terminal residues that meet in the middle, the above closure condition could not be used for this algorithm. Instead, we checked the bond lengths between the backbone atoms belonging to the three central residues in each loop. Any bond length that differed by more than ±0.25 Å from the standard value was discarded. Standard bond lengths were obtained from the published values of Engh and Huber.^28^

A van der Waals (VDW) clash filter was applied to all closed loops. The VDW clash filter uses a three-dimensional grid to screen all loop atoms for clashes with the protein body in linear time. We used the ratio of the distance between two nonbonded atoms to the sum of their van der Waals radii (taken from the XPLOR-NIH^29^ implementation of the CHARMM22 force field) to define a filter. Any loop conformation that contained an atom for which this ratio is smaller than a defined cutoff was rejected. Since many of the loop conformations contained moderate clashes that could be fixed using energy minimization, we used a lenient cutoff of 0.5 so that any two atoms would be allowed to approach each other to within half the sum of their van der Waals radii.

To determine how each method would perform in the context of a real loop prediction strategy, we defined a measure of efficiency as the time in minutes, *T*_usable_, required to generate 10,000 closed loop conformations that do not contain steric clashes (which we term “usable” loops). *T*_usable_ is given by:

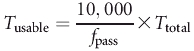
(1)
where *f*_pass_ is the fraction of loops that are closed and not rejected by the VDW clash filter and *T*_total_ indicates the total time required to both close and screen a loop conformation. To calculate *T*_total_, we added the values for the average closure time over 100 loop conformations for each algorithm at each loop length to the average time required to screen a loop conformation for clashes. Since PLOP-build uses a different strategy for loop closure, we obtained closure times for this algorithm by dividing the time to generate all the loop conformations (which can vary from one loop target to the next—see previously) by the total number of loop conformations. Screening times for PLOP-build were obtained by taking the average time to screen all closed loop conformations at each loop length.

### Scoring functions

Loop conformations with added side chains were evaluated with the RAPDF^18^ and DFIRE^22^ statistical potentials and a simplified energy function used in LOOPY. The RAPDF potential was obtained from http://software.compbio.washington.edu/ramp/ramp.html. The DFIRE potential used here was an in-house version of the published potential^22^ that was rederived using a recent high-resolution protein structure data set.^30^ The softened van der Waals potential in LOOPY [see Eq. (10) from Xiang *et al*.^8^] was also tested. This empirical scoring function accounts in a rough way for van der Waals interactions, hydrogen bonding, and hydrophobicity. Its functional form is:

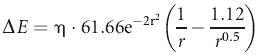
(2)
where *r* is the ratio of the distance between two nonbonded atoms to the sum of the van der Waals radii taken from the CHARMM22^31^ force field, η is a parameter used to account for hydrogen bonding and hydrophobic energy that is scaled according to atom charge, polarity, and the sign of the energy.^26^ If two atoms are negatively and positively charged, η is set to 1.25 or 0.75 depending on whether Δ*E* is negative or positive. Similarly, if the two atoms are both nonpolar, η is set to 1.25 or 0.75 depending on whether Δ*E* is negative or positive. If two atoms are polar and nonpolar, η is set to 0.85 to penalize the interaction. We denote this form of LOOPY's energy function as “LOOPY-sVDW+” where “s” indicates the use of a softened van der Waals expression and “+” indicates that hydrogen bonding and hydrophobicity are implicitly incorporated into the van der Waals expression. The original version of LOOPY used a more detailed scoring function than that given in Eq. (2) and included a surface area-dependent hydrophobicity term and an explicit hydrogen bonding potential. The previous procedure is significantly slower than the one based on Eq. (2) and the results obtained are only marginally improved. It should be noted that while the colony energy^8^ is used during the generation of the loop ensembles, this option is turned off when scoring the ensembles with LOOPY-sVDW+.

## RESULTS

### Assessing the performance of loop closure methods

We evaluate CCD, Wriggling, Random Tweak, Direct Tweak, LOOPY_bb_, and PLOP-build in terms of the computer time required to generate sterically reasonable closed loop conformations. These evaluations are carried out on loops that do not include side chain atoms.

We first generate ensembles of open-loop conformations for a subset of the 8, 11, and 12-residue loop targets considered later on in this study. Specifically, we considered 53 eight, 17 eleven, and 10 twelve-residue loop targets (see supplementary materials for details). In each case the N-terminus of the loop was anchored and φ ϕ, angles were randomly selected from a backbone conformer library.^8^ Five thousand open loop conformations were generated for each of the 8, 11, and 12-residue loop targets. PLOP-build does not permit the number of desired closed loops to be specified in advance since this value is controlled by the sampling resolution. However, PLOP-build does permit the minimum number of loops that will be generated to be specified in advance. Thus, we set the minimum number of loops output by PLOP-build to 5000 for the 8, 11, and 12-residue loop targets.

Table II summarizes a number of performance characteristics of the various loop closure methods. All methods succeeded in closing 90% or more of the open loop conformations (data not shown). All methods except Direct Tweak do not account for interactions between loop atoms and the rest of the protein during the course of the closing procedure. For this reason, most of the loops do not pass the steric filter and would have to be discarded at the next step of a loop prediction protocol. There is little difference between the various procedures (other than Direct Tweak and LOOPY_bb_) in terms of the fraction of loops that pass the filter, ƒ_vdw_, or in terms of the value of RMSD_min_, the closest structure to native that is generated. For the 11 and 12-residue loops, only Direct Tweak and LOOPY_bb_ generate structures with RMSD_min_ values below 2 Å.

**Table II:** Performance Characteristics of Loop Closure Procedures

	Loop lengths					
	8		11		12	
Algorithm	f_VDW_^a^	RMSD_min_^b^	f_VDW_	RMSD_min_	f_VDW_	RMSD_min_
Random Tweak^c^	0.19	1.22	0.06	2.22	0.06	2.64
CCD^d^	0.17	1.20	0.05	2.11	0.05	2.57
Wriggling^e^	0.14	1.43	0.03	2.24	0.04	2.68
PLOP-build^f^	0.17	0.99	0.02	2.18	0.01	2.69
Direct Tweak^g^	0.82	0.69	0.74	1.20	0.78	1.48
LOOPY_bb_^h^	0.83	0.89	0.66	1.51	0.69	1.80

a Fraction of closed and sterically feasible loop conformations.

b RMSD averaged over loop conformations from each ensemble with the smallest RMSD to native.

c Implementation of Xiang *et al*.^8^,^26^

d Implementation of Zhu *et al*.^30^

e In-house implementation of the Wriggling algorithm.^25^

f Dihedral angle based build up procedure of Jacobson *et al*.^9^ obtained from the author.

g Implementation of Xiang *et al*.^8^,^26^

All methods are quite fast and loop closure does not appear to be a rate-limiting step in loop prediction. The times reported for CCD are longer than those for the other algorithms but this may be due to limitations in our local implementation of CCD. Indeed an implementation we obtained from the Dunbrack lab is about seven times faster than our own. CCD has an advantage of algorithmic simplicity and indeed we have recently used it in a study of protein segment refinement.^30^

Direct Tweak and LOOPY_bb_ are much slower than most of the other loop closure algorithms but, since they account for interactions between the loop and the rest of the protein as part of the closure procedure, most of the structures they generate pass the steric filter. In addition the RMSD_min_ values of these conformations are significantly smaller than those of the other algorithms. To compare all algorithms on an equivalent footing, we summarize in Table III the estimated time required to close 10,000 loops that pass the steric filter. Here the performance of Direct Tweak and LOOPY_bb_ are in the range of the other methods but they offer the advantage of producing loop conformations with lower RMSD_min_. Of course the greater efficiency of the other loop closure algorithms suggests that one could use them to generate a much larger number of conformations than generated with Direct Tweak so as to arrive at comparable values of RMSD_min_. However, we have found (data not shown) that this would require generating about a million conformations for 8-residue loops and many more for longer loops. Any loop prediction procedure would then have to add side chains to each of these loops and evaluate them with some scoring function. Thus, the use of Direct Tweak appears to provide a far more effective strategy.

**Table III:** Estimated Time in Minutes Required to Generate 10,000 Closed and Sterically Feasible Loop Conformations

	Tusable^a^		
Algorithm	Eight	Eleven	Twelve
Random Tweak	1.99	8.47	10.17
CCD^b^	159.46	511.10	527.77
Wriggling	5.67	28.50	22.50
PLOP-build	3.39	35.00	71.67
Direct Tweak	34.00	73.44	75.65
LOOPYbb	22.86	62.21	59.15

a See Equation 1.

b The implementation of Canutescu and Dunbrack^24^ is about seven times faster.

As mentioned earlier the LOOPY_bb_ algorithm to generate sterically reasonable closed loops exploits both Random Tweak and Direct Tweak.^8^,^26^ As can be seen in Tables II and III, the performance of LOOPY_bb_ is comparable with that of Direct Tweak. It seems clear that the ability of Direct Tweak to perform an energy minimization in torsion space while accounting for interactions within the entire protein is responsible for its success, whether or not the starting conformation is generated randomly, or with a fast loop closure algorithm.

### Scoring loop ensembles with simple scoring functions

A full loop prediction protocol requires the addition of side chains and a subsequent ranking with some scoring function. Of the methods summarized in Table I, LOOPY is the most efficient and is reasonably accurate as well. Moreover, as can be seen in the previous section, it is based on a particularly efficient approach to loop closure. However LOOPY uses a heuristic scoring function which may not be optimal in terms of its ability to identify native-like conformations. In Table IV LOOPY's scoring function is compared with two widely used statistical potentials, DFIRE and RAPDF, in terms of their ability to rank the native conformation as the best among a LOOPY-generated decoy set. These sets included 1000 conformations for eight-residue loops, 2000 for nine-residue loops, 5000 for ten, eleven, and twelve residue loops, and 8000 for thirteen-residue loops. Loop ensembles are generated using LOOPY for each loop target and then the different energy functions are used to score each conformation, including the native. It is clear that DFIRE is significantly more successful than the other methods in identifying the native conformation.

**Table IV:** Numbers of Cases Where Scoring Functions Rank the Native Loop as Lowest in Energy for Loop Ensembles Generated With LOOPY

		Scoring functions		
Loop length	*N*^a^	DFIRE^b^	LOOPY-sVDW+^c^	RAPDF^d^
8	63	48	18	17
9	56	37	26	20
10	40	28	18	10
11	54	35	26	13
12	40	28	23	13
13	40	32	23	8

a Number of loop targets studied.

b Zhu *et al*.^30^ implementation of the DFIRE statistical potential.

c Modified softened van der Waals scoring function.^26^

d RAPDF^18^ statistical potential.

**Figure 1:**
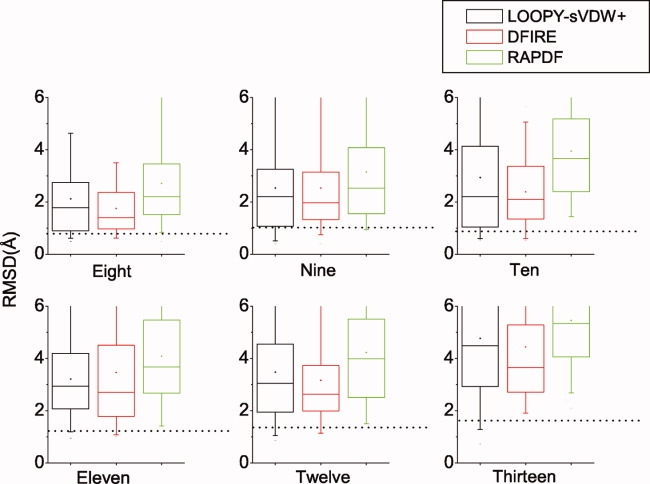
Box plot for various RMSD values obtained from different scoring functions. See text for details.

Of course, in a real modeling application the native structure is not available so that a more important test of a scoring function is how well it selects low RMSD conformations from an ensemble of conformations generated by a loop closure method. In Figure 1 we show box plots to indicate how well each scoring function succeeds in selecting low RMSD conformations from a set of LOOPY-generated loops. The top of each vertical line shows the RMSD of the worst prediction of a given scoring function and the point on the bottom shows the best prediction. The bottom and top horizontal line on each box shows the RMSD of the 25th and 75th percentile prediction, respectively, while the line through the middle shows the median. The average RMSD prediction accuracy for each scoring function is displayed on the graph as a point inside the box. It is clear from the figure that DFIRE is the best of the three scoring functions tested. Its best predictions are almost universally better than those of the other functions and the range of RMSD values within the box tends to be smallest, that is it makes fewer bad predictions. RAPDF appears to be the least effective of the three scoring functions, at least on the loop test set generated here.

**Figure 2:**
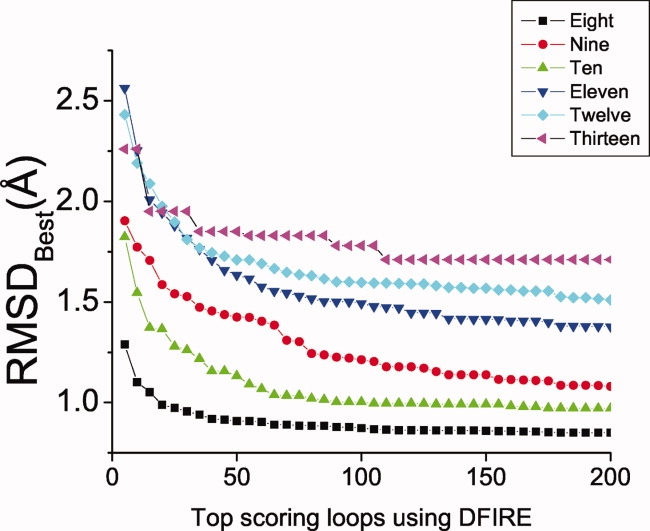
The lowest RMSD to native conformation as a function of the number of top scoring loops (RMSD_Best_) according to DFIRE. The curves represent averages taken over each loop length.

The dashed line in Figure 1 shows the average value of RMSD_min_ for each loop set. As can be seen, most RMSD_min_ values are below 1.5 Å whereas the majority of the RMSD values for conformations selected by the scoring functions are above this value, even when DFIRE is used. Thus, there is significant room for improvement in terms of the consistent selection of low RMSD conformations. One approach is to use more accurate scoring functions, for example from atomic level force fields that include solvation effects. However, these tend to be too slow and too sensitive to small structural variations to apply to a large ensemble of conformations. Figure 2 contains a plot of RMSD_Best_, the average value of the lowest RMSD conformation among the N top scoring loops ranked by DFIRE, as a function of N. For all loop lengths, the plots appear to level off at about 50–100 low-energy loops. This suggests that it might be productive to apply a detailed potential function to a subset of loops that have been selected by a more simplified scoring function. This approach is the basis of the hybrid loop prediction protocol that is described in the next section.

### LoopBuilder

The general protocol described in this section includes: (1) The generation of an ensemble of closed loop conformations with side chains added; (2) Filtering the ensemble with a simple scoring function and retaining only the N top scoring loop conformations; and (3) Using an all atom energy function to minimize and then to rank these N conformations.

On the basis of speed of the Random Tweak algorithm used in LOOPY, and the success of Direct Tweak and LOOPY_bb_ in generating conformations with low values of RMSD_min_, we have adopted the complete LOOPY strategy to obtain an ensemble of starting conformations. Specifically, we use LOOPY_bb_ to generate backbone conformations, add side chains with a modified version of the SCAP^27^ algorithm, and then carry out a torsional energy minimization with Direct Tweak As earlier, the number of closed loop conformations generated was 1000 for eight-residue loops, 2000 for nine-residue loops, 5000 for ten, eleven, and twelve-residue loops, and 8000 for thirteen-residue loops. We have found that increasing the ensemble size to 10,000 leads only to a marginal improvement in accuracy for all loop lengths. We retain 50 loop conformations from each ensemble for further analysis based on the results in Figure 2.

Each of the top 50 conformations was subjected to an energy minimization is Cartesian space using the PLOP program (Version 12).^9^ PLOP uses the OPLS-AA force field and a surface generalized Born solvation model with a nonpolar estimator (OPLS/SBG-NP). We used 1000 steps of truncated Newton energy minimization (or until an RMS gradient of 0.001 kcal/mol/Å was reached). Only atoms in the loop were allowed to move. Energy evaluations were carried out using an internal dielectric constant of 1 and an external dielectric constant of 80.

Table V contains average and median prediction accuracies over the entire set of loop targets using different approaches to ranking. It is clear from the table that the results using LoopBuilder are significantly better than those obtained from LOOPY or from a ranking with DFIRE alone. Moreover, the results obtained by minimizing the top conformations ranked by DFIRE are significantly better than those obtained by carrying out a molecular mechanics energy-minimization on the 50 top conformations ranked by LOOPY. This shows the value of using a more accurate scoring function at the loop filtering stage.

**Table V:** Average and Median Prediction Accuracies Using Loop Ensembles Generated With LOOPY

	Average (median) prediction accuracy			
Loop length	LOOPY^a^	LOOPY/PLOP^b^	DFIRE^c^	Loop builder^d^
8	1.89 (1.59)	1.96 (1.72)	1.69 (1.40)	1.31 (0.97)
9	2.71 (2.04)	3.67 (3.69)	2.52 (1.97)	1.88 (1.17)
10	2.42 (2.18)	3.40 (3.16)	2.41 (2.22)	1.93 (1.64)
11	3.02 (2.48)	4.36 (3.66)	3.43 (2.68)	2.50 (1.95)
12	3.15 (2.71)	4.11 (3.95)	3.15 (2.74)	2.65 (2.41)
13	4.44 (3.46)	5.84 (5.68)	4.35 (3.63)	3.74 (2.85)

a LOOPY prediction.

b Prediction based on a PLOP energy minimization of the 50 low energy loop conformations according to LOOPY.

c Prediction based on a DFIRE ranking of the loops generated using LOOPY.

d Prediction obtained from LoopBuiulder which applies a PLOP energy minimization to the 50 low energy loop conformations selected by DFIRE.

The predictions of LoopBuilder are clearly superior to those reported in Table I, with the exception of PLOP. The average execution time of the hybrid approach over 30 eight-residue loop targets was less than 1 h on a single-dual Xeon processor operating at 1.4 GHz. The average time for 10 twelve-residue loop targets was less than 4.5 h. Thus, LoopBuilder is about three times slower than LOOPY but yields predictions with significantly improved accuracy.

To better simulate the problem of predicting loop conformations in a homology modeling application, we have assumed knowledge only of the backbone conformations and have used the SCAP^27^ program to add side chains to all residues in all of the proteins studied in this article. We then use LoopBuilder to predict the loop conformation over the entire set of targets. In Table VI we compare these results with those obtained when side chains are fixed in the native conformation. Two sets of ensemble sizes were used so as to determine if larger sampling improved results. As can be seen from the table, there is some degradation of the quality of the results when side chains are repacked relative to the results obtained using X-ray coordinates for all side chains. In addition, the table shows that when side chains are repacked, increasing the numbers of loops that are generated offers a significant improvement in prediction accuracy. Using the larger ensemble size increases the computational cost by about a factor of 4 (data not shown).

**Table VI:** Average and Median Loop Prediction Accuracies Obtained With Loop Builder Using Both Native and Repacked Side Chains

	Average (median) prediction accuracy		
Loop length	Native^a^	Repack^a^	Repack^b^
8	1.31 (0.97)	1.37 (1.17)	1.17 (0.79)
9	1.88 (1.17)	1.99 (1.53)	1.69 (0.91)
10	1.93 (1.73)	2.22 (1.90)	1.82 (1.48)
11	2.50 (1.95)	2.94 (2.69)	2.52 (2.28)
12	2.65 (2.41)	3.21 (2.81)	2.71 (2.28)

a Ensemble sizes of 1000 for eight, 2000 for nine, and 5000 for ten, eleven, and twelve-residue loops.

b Ensemble size of 10,000 loop conformations was used for all loop lengths.

Rohl *et al*.^13^ used Rosetta to carry out predictions on a set of 8 and 12-residue loop targets using native protein structures with repacked side chains and they obtained prediction accuracies of 1.45 and 3.62 Å for a set of 8 and 12-residue loops, respectively (see Table I). As can be seen in Table VII, application of LoopBuilder to this data set* yielded similar average RMSDs for 8-residue loops of 1.63 and 1.35 Å using ensemble sizes of 1000 and 10,000, respectively. For 12-residue loops the corresponding numbers are 3.70 and 3.54 Å for ensemble sizes of 5000 and 10,000, respectively. Thus, the performance of LoopBuilder is essentially equivalent to that of Rosetta when both are applied to the modified Rohl *et al*.^13^ dataset but the computational cost of LoopBuilder is likely to be significantly less since it does not involve an extensive Monte Carlo procedure.

**Table VII:** Prediction Accuracies for 8 and 12-Residue Data Set of Rohl et al. Using LoopBuilder With Repacked Side Chains

	Average (median) prediction accuracy		
Loop length	LoopBuilder^a^	LoopBuilder^b^	Rohl *et al*.^c^
8	1.63 (1.14)	1.35 (0.99)	1.46 (1.20)
12	3.70 (2.77)	3.54 (3.11)	3.56 (3.28)

a Ensemble size of 1000 for eight and 5000 for twelve-residue loops.

b Ensemble size of 10,000.

c Average and median prediction accuracies for Rohl *et al*.^13^ were computed over the same set of loop targets considered in Columns 2 and 3.

## DISCUSSION

In this article, we have studied a number of aspects of the loop modeling problem with the goal of developing a computationally efficient protocol for the prediction of loop conformations that can be easily modified and improved. To this end, we have investigated two issues that are common to many problems in protein structure prediction, sampling and scoring. Most approaches to loop modeling begin with the generation of a large number of loops and then use some scoring function to select those that are energetically favorable. If there were a method available that could refine structures from conformations that are far from native, then sampling would not be so important. However the current reality is that many scoring functions do a good job in identifying native-like conformations if they are sampled, but that refinement from conformations that are not very close to the native does not at this stage offer a general purpose solution to the problem.

For this reason, it is important to determine how successful a particular sampling method will be in generating native-like conformations and then to ask whether a particular scoring function will be able to identify these conformations. For the specific case of loop modeling with *ab-initio* methods, which is the subject of this work, we have tested the ability of loop closure methods to generate native-like conformations. Most of the methods that ignore steric clashes perform comparably in terms of speed in the sense that the loop closure step is not rate-limiting in the context of the entire loop prediction protocol. Direct Tweak is a method that generates closed-loop conformations while accounting for interactions between the loop and the rest of the protein. It offers significant improvement in the RMSD_min_ values of the conformations it generates and, in addition, the entire distribution is shifted towards conformations with lower RMSD. This is hardly surprising since a large fraction of the conformations generated by the other methods are not sterically feasible. Thus, one might, in principle, expect that using a faster method which, for the same amount of computer times allows the generation of many more conformations than does Direct Tweak, would yield comparable RMSD_min_ values. However, as pointed out above, an unacceptably large number of conformations would have to be generated with other methods for them to be competitive with LOOPY_bb_ in terms of generating low RMSD_min_ conformations.

As in previous work,^30^ we have found that DFIRE is a particularly effective statistical potential both in terms of its ability to identify native-like conformations, and in the fact that its use as a filter enriches ensembles with conformations with lower RMSD values than the two other scoring functions we tested (Fig. 1). One expects then that any improvement in the development of fast scoring functions will lead to improvements in loop prediction accuracy. The strategy of filtering conformations with a statistical potential and then carrying out a refinement with an MM force field,^9^ is found to be quite effective. In principle, one could just ignore the filtering step and carry out molecular mechanics energy minimizations on all the conformations generated with a given loop closure procedure. For example, we have found that minimizing all conformations belonging to the 8-residue LOOPY-generated ensembles (i.e., >60,000 loops), yields an average RMSD prediction of 1.36 Å. LoopBuilder yields an average RMSD prediction over the same set of loop targets of 1.31 Å. For the 9 and 10-residue loops, energy minimization of all the LOOPY-generated conformations results in an average RMSD prediction of 2.31 and 2.08 Å. In comparison, LoopBuilder yields an average RMSD prediction of 1.88 and 1.93 Å for the 9 and 10-residue loops, respectively. It thus appears that the filtering step with DFIRE somewhat improves prediction accuracy, and of course it reduces the computational cost of the entire loop prediction process by orders of magnitude. Apparently, “turning on” a molecular mechanics force field at too early a stage in the protocol produces incorrect local minima that can be filtered out with DFIRE.

LoopBuilder is similar in many ways to the procedure reported by de Bakker *et al*. which uses RAPPER to generate loop conformations, RAPDF as a filter and all-atom molecular mechanics energy minimization with the AMBER force field with a continuum treatment for the solvent.^15^ The reported prediction accuracy using the RAPPER-based procedure is 2.28–4.99 Å for the Fiser *et al*.^8^–^12^ residue loop targets.^14^ Our results for a different set of loops of comparable length range between 1.31 and 2.65 Å. It is possible that much of the difference between the two methods is due to the apparent superiority of DFIRE over RAPDF (see e.g., Table IV and Ref.^16^).

The combined use of a filtering step followed by a molecular mechanics-based energy minimization appears to be an effective general strategy for structure refinement. We have recently described an iterative, modular optimization (IMO) procedure, for the refinement of protein segments containing secondary structure elements.^30^ IMO also filters conformations with DFIRE and then subjects them to an MM energy minimization step. We have found, in agreement with Zhu *et al.*, that varying DFIRE parameters can affect filtering performance.^30^ However, after energy minimization, the average RMSD over loop sets is fairly insensitive to the specific DFIRE parameterization that is used.

In terms of performance, LoopBuilder offers significant improvement in accuracy over the methods summarized in Table I, with the exception of PLOP^9^,^12^ which, as pointed out above, accounts for crystal contacts. A comparison of Table I to Table V reveals that the results of Jacobson *et al.*^9^ are about 0.3–0.9 Å more accurate than the corresponding results obtained using LoopBuilder depending on loop length and ensemble size. Results from Zhu *et al.* (i.e., PLOP II) are clearly superior to those obtained using LoopBuilder. However, obtaining results of this quality can require weeks of computer time on a single processor. Some of the discrepancy in accuracy is due to the inclusion of the crystal environment and some of it may be due to the extensive hierarchical refinement procedure in PLOP that provides an effective means for densely sampling the conformational space of a loop using a detailed all-atom energy function. In addition, the inclusion of the recently developed hydrophobic contact potential significantly improves the prediction accuracy for longer loops.^12^

There are many practical applications for a fast and accurate loop prediction methodology. In cases where one is interested in finding as accurate a conformation as possible for a particular loop, as in structure-based drug-design, computer time is not necessarily an issue. Thus, methods like PLOP may be the most appropriate. However there are many cases where speed is an issue. For example, when trying to score alternate template-based models for a given protein, it is essential that the loop regions be refined in a consistent way; otherwise there may be a bias towards a particular model simply because the loops were better built in that model. A fast and accurate loop prediction methodology avoids this problem. Moreover, there may be no point in applying a slow method that involves an extensive sampling procedure when there is uncertainty as to the conformation of the rest of the protein, as there often is in homology modeling. In such cases one is generally interested in generating as accurate a loop conformation as possible with a method that does not significantly extend the computation time required for the construction of the entire model. LoopBuilder seems ideally suited for such applications. Moreover, when used in conjunction with related methods, such as our IMO procedure, that sample and score regions of proteins that contain secondary structure elements, it is possible to develop a local refinement procedure for homology models that focuses on regions of a protein whose conformations are most uncertain. These in turn might be identified based on sequence alignments, or from some local scoring function that identifies regions that appear to be energetically unfavorable.

## Footnotes

The following loops belonging to Rohl *et al.* data set were not considered because they were either obsolete entries in the PDB (4fxn, 3b5c, and 4ilb) or we could not energy minimize the loop (1hfc, 1lst).
